# *GhWRKY40*, a Multiple Stress-Responsive Cotton WRKY Gene, Plays an Important Role in the Wounding Response and Enhances Susceptibility to *Ralstonia solanacearum* Infection in Transgenic *Nicotiana benthamiana*

**DOI:** 10.1371/journal.pone.0093577

**Published:** 2014-04-18

**Authors:** Xiuling Wang, Yan Yan, Yuzhen Li, Xiaoqian Chu, Changai Wu, Xingqi Guo

**Affiliations:** State Key Laboratory of Crop Biology, College of Life Sciences, Shandong Agricultural University, Taian, Shandong, PR China; ISA, Portugal

## Abstract

WRKY transcription factors form one of the largest transcription factor families and function as important components in the complex signaling processes that occur during plant stress responses. However, relative to the research progress in model plants, far less information is available on the function of WRKY proteins in cotton. In the present study, we identified the *GhWRKY40* gene in cotton (*Gossypium hirsutum*) and determined that the GhWRKY40 protein is targeted to the nucleus and is a stress-inducible transcription factor. The *GhWRKY40* transcript level was increased upon wounding and infection with the bacterial pathogen *Ralstonia solanacearum*. The overexpression of *GhWRKY40* down-regulated most of the defense-related genes, enhanced the wounding tolerance and increased the susceptibility to *R. solanacearum*. Consistent with a role in multiple stress responses, we found that the *GhWRKY40* transcript level was increased by the stress hormones salicylic acid (SA), methyl jasmonate (MeJA) and ethylene (ET). Moreover, GhWRKY40 interacted with the MAPK kinase GhMPK20, as shown using yeast two-hybrid and bimolecular fluorescence complementation systems. Collectively, these results suggest that *GhWRKY40* is regulated by SA, MeJA and ET signaling and coordinates responses to wounding and *R. solanacearum* attack. These findings highlight the importance of WRKYs in regulating wounding- and pathogen-induced responses.

## Introduction

Stress is perceived and transduced through a series of signaling molecules that ultimately affect the regulation of stress-inducible genes to initiate the synthesis of different types of proteins, including transcription factors, enzymes, molecular chaperones, ion channels, and transporters, or to alter their activities [1]. Among these proteins, transcription factors (TFs) are crucial in eliciting stress responses by modulating the expression of specific target genes in a temporal and spatial manner; they are also necessary for normal development and proper responses to physiological or environmental stimuli [2]–[5]. WRKY proteins are a class of zinc finger-containing TFs that are encoded by large gene families in all higher plants and are reported to play a pivotal role in many physiological processes. WRKY TFs share a highly conserved sequence of approximately 60 amino acids called the WRKY domain, which contains the conserved amino acid sequence motif WRKYGQK at the N-terminus and a novel zinc finger-like motif at the C-terminus. Based on their domain structures, WRKY TFs are classified into three major groups (I, II, and III) [6]. Additionally, WRKY TFs act as transcriptional regulators by binding to the W-box, which is present in the promoter regions of various stress-related genes, thus regulating the expression of many genes, resulting in stress tolerance [7].

WRKY TFs have mostly been studied with respect to their participation in the regulation of defense against biotic stresses or against tolerance of abiotic stresses [8]. Some WRKY proteins are reported to be involved in the coordination of multiple biological processes. For example, *AtWRKY33* regulates disease resistance, NaCl tolerance and thermotolerance [9]–[11], while Ca*WRKY40* modulates tolerance to heat stress and resistance to *Ralstonia solanacearum* infection [12]. This suggests that some WRKY proteins serve as nodes in a crosstalk between different physiological processes. However, the functions of the majority of WRKY family members and their possible roles in signaling crosstalk are limited.

Previous studies have shown that several wound-responsive WRKY genes are also regulated by pathogen infection [13]–[16]. These reports have provided useful research methods and broadened our knowledge on the function of plant WRKYs. However, so far, few reports have addressed the mechanistic details of the crosstalk between wounding and pathogen infection. The prominent role of WRKYs in stress signaling indicates a promising target for applied studies in crop species. Moreover, dissecting the crosstalk between different pathways is critical to understanding the plant response to environmental cues. Wounding presents a threat to plant survival because it not only physically destroys plant tissues but also provides a pathway for pathogen invasion. Therefore, it is necessary to map the interaction between the wounding response and pathogen infection and to identify novel genes involved in these processes. In particular, the molecular mechanisms involved in these processes should be examined in different genetic backgrounds.

WRKY proteins have been linked to the MAP kinase (MAPK) cascade in *Arabidopisis*; *AtWRKY22* and *AtWRKY29* are thought to function downstream of *MPK3/MPK6* [17]. The MAPK cascade is the basic module for transmitting signals from upstream ligand receptors to downstream substrates in response to various biotic and abiotic stress signals, hormones, and growth and developmental processes [18]. In tobacco, WRKY8 is a physiological substrate of SIPK, NTF4, and WIPK [19]. In rice, OsWRKY30 interacts with and is phosphorylated by OsMPK3 [20]. Thus, only a limited number of upstream WRKY components have been identified, particularly in crops, and whether MAPKs interact with WRKYs in cotton should be investigated.

Cotton is one of the most economically important crops worldwide and is an excellent source of fiber and oil. However, its growth and yield are severely inhibited under various biotic and abiotic stress conditions. The applied study of cotton WRKYs will provide new insight that may aid in creating cotton plants that are better able to adapt to environmental challenges. However, the majority of WRKY TFs in cotton have not been characterized. In the present study, a group II a WRKY gene from cotton (*Gossypium hirsutum*), *GhWKRY40*, was isolated and characterized. *GhWRKY40* expression was induced by various abiotic and biotic stresses. We obtained information on the ability of *GhWRKY40* overexpression to alter the responses to wounding and infection with the bacterial pathogen *R. solanacearum* in *Nicotiana benthamiana*. Moreover, we demonstrated that GhWRKY40 interacted with GhMPK20 but not GhMK6a, two MAPKs that were previously identified by our group, using a yeast two-hybrid system and bimolecular fluorescence complementation (BiFC). This study suggests that the transcriptional responses of *GhWRKY40*-overexpressing plants to wounding may be related to defense signaling pathways.

## Materials and Methods

### Cloning of the full-length *GhWRKY40* cDNA

Total RNA was extracted from the leaves of seven-day old cotton seedlings using a modified cetyltrimethylammonium bromide (CTAB) protocol [21]. Reverse transcription-PCR (RT-PCR) and RACE-PCR were used to amplify the full-length *GhWRKY40* cDNA. A pair of degenerated primers (MP1/MP2) was designed to isolate WRKY family members from the cotton cotyledons. According to the obtained fragment, specific primers (5P1/5P2, 3P1/3P2, and QC1/QC2) were used for 5′ rapid amplification of cDNA ends (RACE), 3′ RACE and the identification of the full-length cDNA sequence. The general PCR procedures and primers are shown in Table S1 and Table S2, respectively. The PCR product was purified, cloned into the pEasy-T1 vector, and transformed into competent *Escherichia coli* cells for sequencing. The amino acid sequence alignment of GhWRKY40 and its homologues was conducted using BLAST (http://www.ncbi.nlm.gov/blast) and DNAman software 5.2.2. The phylogenetic tree was performed in MEGA version 4.1 (http://megasoftware.net) using the neighbour-joining method.

### Amplification of the *GhWRKY40* genomic sequence and promoter

For the amplication of *GhWRKY40* genomic sequence, one pair of primers (QG1 and QG2), which was designed and synthesized based on the full-length *GhWRKY40* cDNA, was used. Genomic DNA was isolated from seedling leaves using the CTAB method. Inverse-PCR (I-PCR) was performed to obtain the promoter sequence. Three restriction endonucleases (NdeI, SspI and VspI) were used to digest the cotton seedling genomic DNA, and T4 DNA ligase was used to self-ligate the DNA fragments into circles, which were used as templates to amplify the promoter region. Three promoter fragments were amplified using six pairs of primers (Nde1/2 and Nde3/4, Ssp1/2 and Ssp3/4, Vsp1/2 and Vsp3/4). The deduced portion of the promoter was subsequently verified using the special primers WP1 and WP2. The sequences of the primers are provided in Table S2. The programme PlantCARE (http://bioinformatics.psb.ugent.be/webtools/plantcare/html) and PLACE (http://www.dna.affrc.go.jp/PLACE/) was used to analyze the *GhWRKY40* promoter sequence.

### Subcellular localization of GhWRKY40

The coding sequence of *GhWRKY40* without the termination codon was amplified by PCR using the primers Wgf1 (5′-GGATCCATGGATACTTCTTCATGGGTGG-3′, *Bam*HI site underlined) and Wgf2 (5′-CTCGAGCTTATAGTTGACAAAATCATAGAAAC-3′, *Xho*I site underlined). Then, the coding sequence was ligated into the binary vector pBI121-GFP, which contains the *Cauliflower mosaic virus* (CaMV) 35S promoter, to yield the expression vector p35S::GhWRKY40-GFP. The resulting expression plasmid, pBI121-GhWRKY40-GFP, or the pBI121-GFP control plasmid was transformed into onion (Allium cepa) epidermis cells via biolistic bombardment transformation using the Biolistic PDS-1000/He system (Bio-Rad, USA) with gold particles (1.0 µl) and a helium pressure of 1,100 psi. And then plated on MS agar medium in the dark condition at 28°C for 24 h, the nuclei were stained with 100 µg/ml of 4′,6-diamidino-2-phenylindole (DAPI) in phosphate-buffered saline for 4 min, and the fluorescence signal of the GhWRKY40-GFP fusion protein was imaged using a fluorescence microscope using excitation wavelength of 488 nm and 350 nm, respectively. The vector p35S::GFP was used as a control.

### Transactivation assay

The transactivation activity of the GhWRKY40 protein was investigated in the Y2HGold yeast strain, which contains the *HIS3*, *ADE2* and *MEL1* reporter genes and the GAL4 promoter. The coding region of *GhWRKY40* was amplified using the primers Wbd1 and Wbd2, which possess *EcoR*I and *BamH*I sites (underlined), 5′-GAATTCATGGATACTTCTTCATGGGTGG-3′ and 5′- GGATCCTTCAACTGGACTTTGCTGAAAC-3′ to build the pGBKT7-GhWRKY40 vector (Clontech, TaKaRa) containing the GAL4 DNA-binding domain. The plasmid pGBKT7-GhWRKY40 and pGBKT7 (negative control) was transformed into Y2HGold yeast cells. The transformed yeast cells were streaked on SD/-Trp and SD/-Trp-Ade-His medium plates to observe yeast growth at 30°C for 3–4 days. An assay of α-galactosidase activity was performed using X-α-gal.

### Cotton growth conditions and *GhWRKY40* expression assay

Cotton (*Gossypium hirsutum* L. cv. lumian 22) seeds were grown under greenhouse conditions at 25±1°C with a 16 h light/8 h dark cycle (relative humidity of 60–75%), and seven-day-old seedlings were used for the following treatments. For the pathogen treatment, cotton seedlings were inoculated with *R. solanacearum* suspensions using the root dip method. For the H_2_O_2_ treatment, cotton seedlings were sprayed with 10 mM H_2_O_2_. The wounding, MeJA, SA and ET treatments were performed as described previously [22]. The treated cotyledons were collected, frozen directly in liquid nitrogen and stored at −80°C for RNA extraction and further analysis. Each treatment was repeated at least three times.

Total RNA was extracted from the treatment samples using a modified cetyltrimethylammonium bromide (CTAB) protocol [21] and then treated with RNase-free DNaseI to remove any potential genomic DNA contamination. First-strand cDNA was synthesized using the EasyScript First-Strand cDNA Synthesis SuperMix. The *GhWRKY40* (KC414679) gene primer pairs WRT1/WRT2 and *Ghubiquitin* (EU304080) primer pairs Ub1/Ub2 were used for quantitative real-time PCR (qPCR) with the SYBR PrimeScript RT-PCR Kit in a CFX96TM Real-time System. The PCR programme was as follows: predenaturation at 95°C for 30 s; 40 cycles of 95°C for 5 s, 55°C for 15 s and 72°C for 15 s; and a melt cycle from 65°C to 95°C. The data was analyzed using the CFX Manager software, version 1.1, and significant differences were determined using the Statistical Analysis System (SAS) software (version 9.1). All reactions were performed with three technical replicates.

### Wounding analysis of transgenic plants

The *GhWRKY40* coding region was amplified with primers WOE1 (5′-GGATCCATGGATACTTCTTCATGGGTGG-3′, *BamH*I site underlined) and WOE2 (5′-GAGCTCTTCAACTGGACTTTGCTGAAAC-3′, *Sac*I site underlined). Then this fragment was subcloned into pBI121 under the control of the CaMV35S promoter. The recombinant plasmid was introduced into the *Agrobacterium tumefaciens* (strain LBA4404) for *Nicotiana benthamiana* (*N. benthamiana*) transformation using the leaf disc method as described previously [23]. The transgenic seedlings were screened on Murashige and Skoog (MS) agar medium containing 100 mg/L of kanamycin and further confirmed by PCR. Eight independent transgenic T_1_ native tobacco lines were obtained. Three independent *GhWRKY40-OE* lines (OE1, OE2 and OE3) and wild-type plants were used for the following experiments. The transgenic T_2_ lines were used in the experiments.

Transgenic *N. benthamiana* seeds were surface sterilized and planted on MS medium for germination under greenhouse conditions. Four-leaf stage seedlings were transplanted into soil and maintained under greenhouse conditions. For the wounding treatment, the third leaf from the top of 8-week old seedlings was cut with scissors. After wounding, the leaves were harvested at the indicated timepoints for histochemical staining and the preparation of RNA. The experiment was repeated at least three times.

After wounding treatment, the OE and WT leaves were incubated with 3,3′-Diaminobenzidine (DAB, 1 mg/mL, pH 3.8) or Nitro Blue tetrazolium (NBT, 0.1 mg/mL) for 36 h at 25°C in the dark. Then, the leaves were boiled in ethanol (95%) for 5 min. After cooling, the leaves were soaked and preserved in fresh ethanol at room temperature and photographed. To examine oxidative tolerance, leaf discs (1.3 cm in diameter) were detached from healthy, fully expanded leaves of OE and WT plants, floated in methyl viologen (MV) (0, 200, 400 or 600 µM) solutions for 64 h, and immersed in 95% ethanol for 40 h to extract chlorophyll for spectrophotometric measurement.

### Disease resistance analysis of transgenic plants

For the disease resistance analysis, *R. solanacearum* strains were cultured at 200 rpm and 37°C in Luria-Bertani (LB) broth. The bacterial cell density was diluted to OD_600_ = 0.6–0.8. A total of 20 µL of the resulting *R. solanacearum* suspension was injected into the third leaf from the top of each plant of 8-week old seedlings using a syringe with a needle. The leaves were harvested at the indicated timepoints for the preparation of RNA. The experiment was repeated at least three times.

### Expression analysis of defense-related genes in transgenic and WT lines

Total RNA of all samples was extracted with TRIzol reagent. qPCR was performed using cDNA as the template, which was synthesized from total RNA extracted from the transgenic or WT *N. benthamiana* lines after wounding and disease treatments. *N. benthamiana β-actin* (*Nbβ-actin*) genes were used as the standard controls. The GenBank accession numbers of the defense-related genes examined in the qPCR analysis are as follows: JQ256516.1 (*β-actin*), ACY30445.1 (*JAZ1*), BAG68657.1 (*JAZ3*), X84040.1 (*LOX1*), AF392978 (*ACS6*), U15933.1 (*APX*), AB093097 (*SOD*), D10524 (*GST*), X12485.1 (*PR1a*) M60460.1 (*PR2*), EH365959.1 (*PR4*), and Y07563 (*HIN1*). The primers of the defense-related genes examined in the qPCR are listed in Table S3.

### Yeast two-hybrid and BiFC assay

For the yeast two-hybrid assay, the *GhWRKY40* cDNA fragment was cloned into the pGADT7 vector in-frame with the GAL4 activation domain. The *GhMPK6a* and *GhMPK20* cDNA fragments were cloned into the pGBKT7 vector in-frame and proximal to the binding domain. These vectors were co-transformed into the Y2HGold yeast strain using the Matchmaker Gold Yeast Two-Hybrid System. Positive clones were plated onto selective SD medium (DDO: SD/-Leu/-Trp, QDO: SD/-Ade/-His/-Leu/-Trp and QDO/X/A: QDO with X-α-gal and aureobasidin A). For the BiFC assay, the *GhWRKY40* and *GhMPK20* cDNA fragments were cloned into pUC-SPYCE-35S and pUC-SPYNE-35S, respectively. These two BiFC constructs were co-transformed into onion epidermal cells using the particle bombardment method, and the fluorescent signal of the resultant proteins was detected using a confocal microscope.

### Statistical analysis

Data were shown as the mean ± standard deviation (SD) with n = 3. The results were analyzed using multiple comparisons by analysis of variance (ANOVA), and means were separated by the Duncan's Multiple Range test. ANOVA was performed using Statistical Analysis System (SAS) version 9.1 software.

## Results

### Characterization of *GhWRKY40*

The full-length cDNA of *GhWRKY*40 was determined to be 1463 bp. It contained an open reading frame (ORF) of 945 bp, encoding 314 amino acids, an untranslated region of 203 bp at the 5′ end and 315 bp of the noncoding region at the 3′ end. The relative molecular mass and theoretical *pI* of the predicted protein were 34.4 kDa and 8.45, respectively. According to the nomenclature for plant WRKYs as well as the alignment this WRKY sequence with related sequences, it was found to share 48.0% and 47.48% identity with AtWRKY40 (NP_178199.1) and BnWRKY40 (ACQ76806.1), respectively. Therefore, we termed the cDNA clone as *GhWRKY40* (GenBank accession number: KC414679).

**Figure 1:**
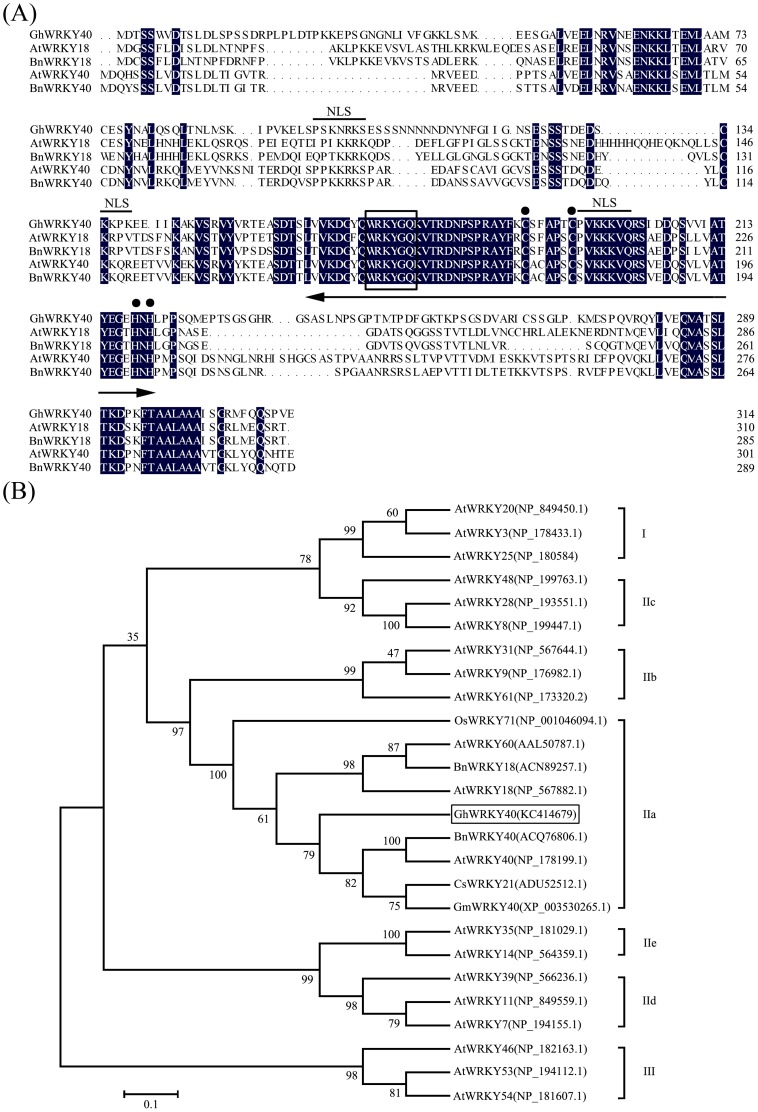
Characterization and sequence analysis of GhWRKY40. (A) Alignment of the amino acid sequences of GhWRKY40 and the representative related proteins AtWRKY18 (NP_567882), BnWRKY18 (ACN89257), AtWRKY40 (NP_178199) and BnWRKY40 (ACQ76806). Amino acids with 100% identity are shaded in black. The approximately 60-amino acid WRKY domain and the C and H residues in the zinc-finger motif (C-X_4–5_-C-X_22–23_-H-X_1_-H) are marked by a two-headed arrow and dot, respectively. The highly conserved amino acid sequence WRKYGQK in the WRKY domain is boxed. The putative nuclear localization signals are marked by lines. (B) Phylogenetic relationship between GhWRKY40 and other plant WRKY proteins. A neighbor-joining phylogenetic tree was created using MEGA 4.1 software. GhWRKY40 is boxed. Each gene name is followed by its protein ID. The abbreviations of the gene names are indicated as follows: Gh, *Gossypium hirsutum*; At, *Arabidopsis thaliana*; Os, *Oryza sativa*; Bn, *Brassica napus*; Cs, *Cucumis sativus* and Gm, *Glycine max*.

According to our multiple alignment and phylogenetic analyses of the WRKY proteins, GhWRKY40 was placed into group IIa of the WRKY superfamily (Fig. 1). The GhWRKY40 protein possesses fully canonical motif structures, including a typical DNA binding domain, the WRKY domain and a putative zinc finger structure (C-X_4–5_-C-X_22–23_-H-X_1_-H). The phylogenetic relationships among various WRKY IIa subgroup members from different organisms were further analyzed by comparing the protein sequences of their conserved WRKY domains.

To analyze the structure of GhWRKY40, we isolated a 1936 bp genomic fragment (GenBank accession number: KC414680) from cotton genomic DNA. Sequence comparison revealed that *GhWRKY40* has four introns. This intron number differs from that of *AtWRKY40* but is similar to that of *AtWRKY18*, *AtWRKY60* and is characteristic of group IIa of the WRKY superfamily.

### GhWRKY40 is localized to the nucleus

**Figure 2:**
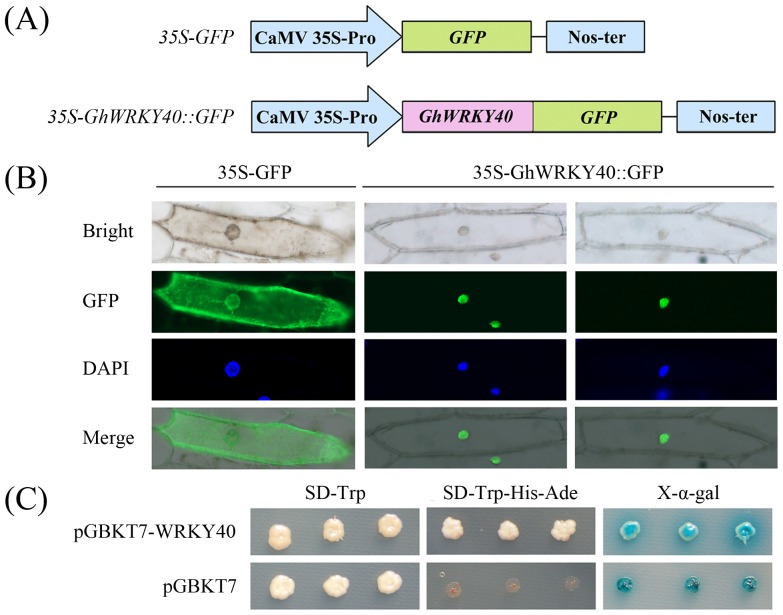
Subcellular localization of the GhWRKY40 protein and transcriptional activation of the *GhWRKY40* gene. (A) Schematic diagram of the 35S-GhWRKY40::GFP fusion protein construct and the 35S-GFP construct. (B) Transient expression of the 35S-GFP and 35S-GhWRKY40::GFP constructs in onion epidermal cells. Green fluorescence corresponding to the expressed proteins was observed with a fluorescence microscope 24 h after particle bombardment. The nuclei of the onion cells were visualized by DAPI staining. (C) Transactivation of the GhWRKY40 gene in yeast. The vector pGBKT7 was used as a control. The transformed yeast culture was streaked onto SD/-Trp or SD/-Trp-His-Ade medium, and the α-galactosidase activity was determined. Three independent experiments were performed.

The PSORT program predicted that GhWRKY40 is localized to the nucleus. Sequence analysis using WoLF PSORT (http://wolfpsort.org/) indicated that the predicted GhWRKY40 protein contains three putative nuclear localization signals (^98^PSKNRKS^104^, ^135^KKPK^138^, ^195^PVKKKVQ^201^; Fig. 1A). To confirm its nuclear localization, we generated a construct for the expression of GhWRKY40 fused to green fluorescent protein (GFP) under the control of the constitutive CaMV35S promoter (Fig. 2A). Typical results indicated the exclusive localization of GhWRKY40-GFP in the nucleus, whereas GFP alone occurred throughout in the cell (Fig. 2B). This result suggests that GhWRKY40 has a nuclear localization.

### GhWRKY40 functions as a potential transcriptional activator

To determine whether the GhWRKY40 protein has transcriptional activity in eukaryotic cells, a plasmid containing the GAL4 DNA binding domain and the whole ORF of *GhWRKY40* was constructed (pGBKT7-GhWRKY40). The plasmid pGBKT7-GhWRKY40 or pGBKT7 (negative control) was transformed into Y2HGold yeast cells. All transformants containing pGBKT7-GhWRKY40 and pGBKT7 grew well on selective medium without tryptophan (SD/-Trp). As shown in Fig. 2C, yeast transformed with pGBKT7-GhWRKY40 grew on selective medium without tryptophan, histidine and adenine (SD/-Trp-His-Ade). In addition, α-galactosidase activity was detected in these cultures, indicating that the expression of the reporter genes (*HIS3, ADE2* and *MEL1*) was activated, whereas neither nor *HIS3* were activated in yeast transformants containing the negative control plasmid (pGBKT7). These results indicated that GhWRKY40 is a transcriptional activator.

### Expression profile of *GhWRKY40* under stress conditions

**Figure 3:**
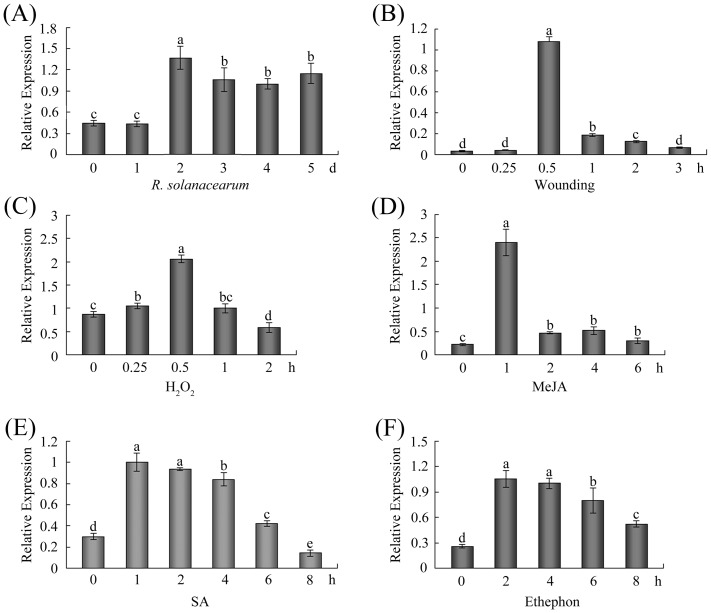
Expression of the *GhWRKY40* gene in response to stress. Seven-day-old cotton seedlings in hydroponic culture were treated with *R. solanacearum* (A), wounding (B), 100 µM H_2_O_2_ (C), 100 µM MeJA (D), 2 mM SA (E) or 5 mM ET released from ethephon (F). Total RNA was isolated at the indicated times after the treatment and subjected to qPCR analysis. The *ubiquitin* gene was employed as an internal control. This experiment was repeated at least twice. The values indicated by the different letters are significantly different at P<0.01, as determined using Duncan's multiple range tests.

To test if GhWRKY40 is involved in the plant response to abiotic and biotic stresses, the transcript levels of *GhWRKY40* were measured by qPCR after the treatment of cotton seedlings with *R. solanacearum*, wounding or H_2_O_2_. The *GhWRKY40* transcript level was up-regulated in response to infection with the bacterial pathogen *R. solanacearum* (Fig. 3A). The *GhWRKY40* transcript level was also increased at 0.5–2 h after wounding, with peak expression 0.5 h (Fig. 3B). During H_2_O_2_ treatment, the *GhWRKY40* transcript level was noticeably elevated at 0.5 h (Fig. 3C). These results suggest that the expression of the *GhWRKY40* gene is induced by environmental stimuli and may play an important role in the stress response.

Phytohormones, such as SA, JA and ET, serve as significant signalling molecules in the regulation of plant defense responses against biotic and abiotic stresses and play vital roles in mediating the expression of downstream defense genes [24]. To determine the possible involvement of *GhWRKY40* in the signaling cascades, the *GhWRKY40* transcript levels were determined by qPCR in seven-day old cotton seedlings that were exogenously treated with MeJA, SA or ET. In response to 100 µM MeJA, the transcript level of *GhWRKY40* was enhanced from 1–6 h, with maximum expression at approximately 1 h (Fig. 3D). *GhWRKY40* mRNA was also induced and reached a peak at 1–2 h with 2 mM SA, (Fig. 3E). Application of 5 mM ET increased the *GhWRKY40* transcript level at 2–8 h, reaching maximal levels from 2–4 h (Fig. 3F). The strong induction of GhWRKY40 expression by these signaling molecules suggests that this gene is involved in signaling pathways in stress resistance.

### *GhWRKY40* promoter analysis

To determine whether the *GhWRKY40* gene is induced by stress, we isolated a 782 bp fragment from the upstream region of the *GhWRKY40* gene by I-PCR. Analysis of this region using the PlantCARE and PLACE databases revealed many putative *cis*-elements, suggesting that *GhWRKY40* plays a role in the plant response to environmental stress. The elements in this region include pathogen/elicitor-related elements, such as ARE, RAV1AAT and WBOXATRNPR1 and WBOX71OS, abiotic stress responsive elements, such as MBS, CCAAT-box, WBOXNTERF3, OSE2ROOTNODULE and CURECORECR, and tissue-specific and development-related elements, such as the circadian, Skn-1 motif, POLLEN1LELAT52 and WBOXHVISO1. All of the identified *cis*-elements are listed in Table 1.

**Table 1:** Putative *cis*-elements in the *GhWRKY40* promoter.

*cis*-element	Position	Sequence (5′-3′)
**Abiotic stress response elements**		
MBS	−116(+)	TAACTG
CURECORECR	−490(+)	GTAC
CCAAT-box	−615(−)	CAACGG
WBOXNTERF3	−304 (−),−372(+)	TGACY
**Pathogen/elicitor response elements**		
ARE	−411(−)	TGGTTT
RAV1AAT	−307(+)	CAACA
WBOXATNPR1	−371(+),−305(−)	TTGAC
WRKY71OS	−55(+),−141(+),−372(+),−305(−)	TGAC
**Tissue-specific and development-related elements**		
circadian	−683(−)	CAANNNNATC
Skn-1_motif	−53(−),−139(−)	GTCAT
OSE2ROOTNODULE	−216(+),−323(−),−530(−),−652(−),−342(−),−352(−)	CTCTT
POLLEN1LELAT52	−1004(+),−828(+),−604(+),−569(+),−271(+),−253(+),−28(+),−9(+),−861(−)	AGAAA
WBOXHVISO1	−372(+),−304(−)	TGACT
**Light regulation elements**		
AE-box	−654(−)	AGAAACAA
GA-motif	−347(+)	AAGGAAGA
I-box	−578(−)	GATAAGAATA
Sp1	−327(+)	CC(G/A)CCC

### Overexpression of *GhWRKY40* in transgenic plants affects the expression of defense-related genes in response to wounding

The *GhWRKY40* transcript patterns suggest a role for this gene in defense against biotic and various abiotic stresses. To assess the significance of this gene in the response to these stresses, we generated transgenic native tobacco T_2_ lines that overexpress *GhWRKY40* driven by the CaMV35S promoter. Except for the later germination of the transgenic plants relative to the WT plants (Fig. S1), we observed no differences between the plants. Wounding is a common plant injury and presents a potential threat to plant survival because it not only damages tissues but also provides means for pathogen invasion. Three independent *GhWRKY40-OE* lines and wild-type plants were used to understand the wounding response.

**Figure 4:**
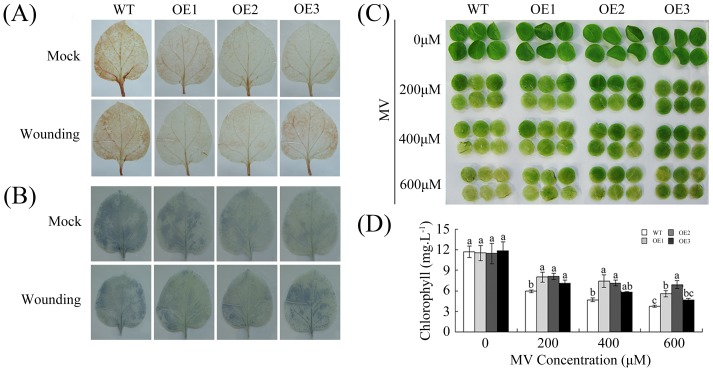
Analysis of ROS accumulation after wounding in WT and OE plants. (A–B) Wound-induced H_2_O_2_ and O_2_^−^ accumulation, as detected via DAB staining and NBT staining, respectively. (C) The phenotype of leaf disks from WT and OE plants that were incubated in different concentrations of MV (0, 200, 400 or 600 mM). (D) Relative chlorophyll content in the leaf disks from (C). Disks floated in water were used as a control. The presented data are the means ± standard error of three independent experiments. The different letters above the columns indicate significant differences (P<0.01) according to Duncan's multiple range test, which was performed using SAS version 9.1 software.

We first examined the wounding response by the DAB staining of H_2_O_2_ accumulation and the NBT staining of O_2_^−^ accumulation in wounded leaves. Compared with WT plants, the *GhWRKY40-OE* lines exhibited clearly decreased DAB staining intensities in their wounded leaves, reflecting low levels of H_2_O_2_ accumulation (Fig. 4A). These lines also exhibited decreased NBT intensities staining in the treated leaves compared with the WT plants (Fig. 4B). Meanwhile, the *GhWRKY40-OE* lines exhibited slightly lower H_2_O_2_ and O_2_^−^ accumulation compared with the WT plants in the absence of wounding treatment. Additionally, leaf discs from the overexpression lines were used to illustrate whether *GhWRKY40* plays a role in oxidative resistance. Leaf discs were soaked in solutions containing various concentrations (0, 200, 400 or 600 µM) of methyl viologen (MV). The results presented in Fig. 4C and 4D show that the transgenic plants exhibited intense oxidative resistance. Leaf discs from both the OE and WT plants showed signs of chlorosis, but neither of these plants showed abnormalities in water. However, MV treatment led to more severe damage in the leaf discs from the WT plants. This result was further confirmed by measuring the leaf disc chlorophyll content before and after MV treatment. Taken together, *GhWRKY40* overexpression appears to enhance the defense response to wounding, resulting in decreased H_2_O_2_ and O_2_^−^ levels.

**Figure 5:**
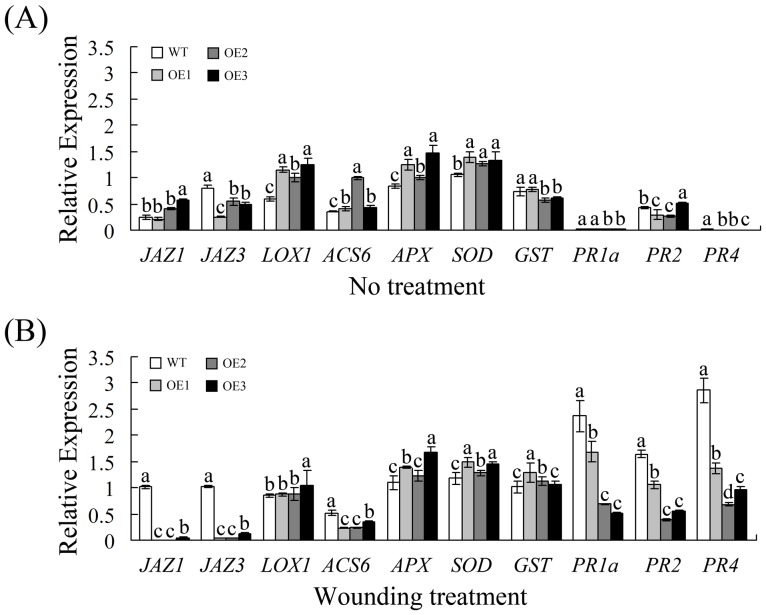
qPCR analysis of stress-related gene expression in WT and OE plants under normal conditions (A) and after wounding (B). The data are presented as the mean ± standard error of three independent experiments. The values indicated by the different letters are significantly different at P<0.01, as determined using Duncan's multiple range tests.

To further confirm the role of *GhWRKY40* in the wounding response and to elucidate its possible mechanism of action, the transcriptional responses of known defense genes to *GhWRKY40* overexpression were investigated by qPCR (Fig. 5). We examined the transcript levels of the JA-responsive genes *JAZ1*, *JAZ3*, and *LOX1*, the ET production-associated gene *ACS6*, the reactive oxygen species (ROS) detoxification-associated genes *APX*, *GST*, and *SOD*, and the SA-responsive genes *PR1a*, *PR2*, and *PR4*. Previous studies have shown that each of the tested genes is up-regulated in response to wounding [16], [25]. We found that the transcript levels of the two *JAZ* genes were clearly decreased in the OE plants after wounding compared with the WT plants. In contrast, we did not find a difference in the *LOX1* transcript level between the WT and OE plants in response to wounding. The transcript expression of the ET production-associated genes, which have been identified as early wound-response genes [26]–[28], was inhibited in the OE plants in response to wounding. Interestingly, *GhWRKY40* does not appear to significantly enhance the expression of *APX*, *GST*, or *SOD*. In the WT plants, the transcript levels of *PR1a*, *PR2*, and *PR4* were up-regulated in response to wounding. However, this induction was attenuated by the overexpression of *GhWRKY40*. Based on the above analysis, it was proposed that wounding may elicit the activation of pathways that interact with the defense response and possibly other signaling pathways. Most importantly, *GhWRKY40* may play a crucial role in the wounding response.

### Overexpression of *GhWRKY40* increases the susceptibility to *R. Solanacearum* in transgenic plants

**Figure 6:**
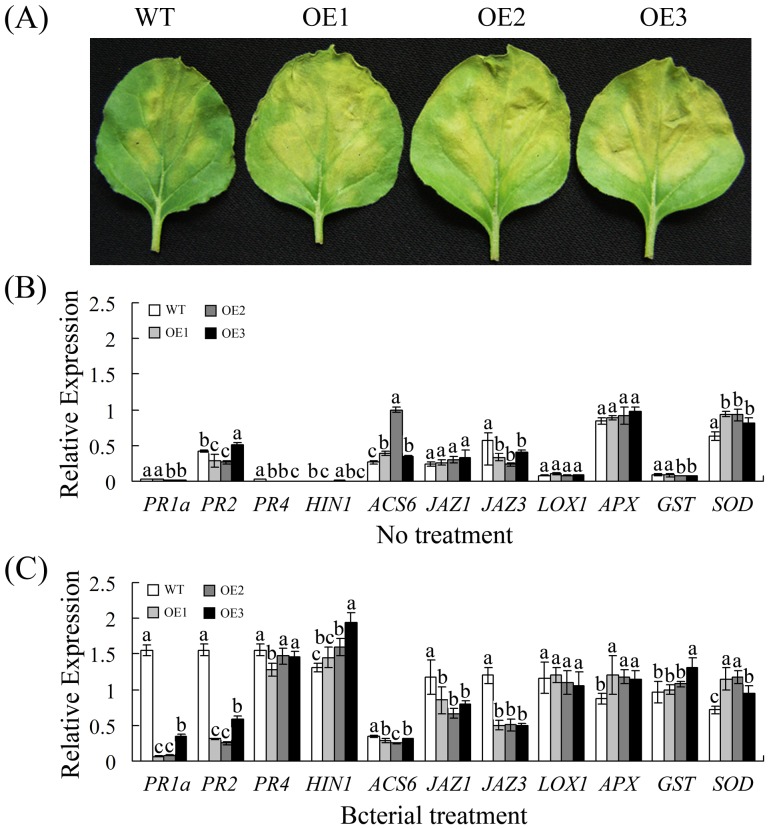
*GhWRKY40* overexpression enhances susceptibility to *R. Solanacearum* in transgenic plants. (A) Phenotype of WT and OE lines after 5 days of incubation with *R. solanacearum*. (B–C) Relative transcript levels of defense-related genes in non-infected and infected WT and OE plants were analyzed by qPCR. The data are presented as the mean ± standard error of three independent experiments. The values indicated by the different letters are significantly different at P<0.01, as determined using Duncan's multiple range tests.

The up-regulation of the *GhWRKY40* transcript in response to *R. solanacearum* suggests a role for this gene in the defense response. To analyze the role of *GhWRKY40* in plant basal defense, the bacterial pathogen *R. solanacearum* was used to infect the OE and WT plants. Five days after infection, all three of the tested transgenic lines exhibited enhanced wilting symptoms and chlorosis (Fig. 6A), indicating that *GhWRKY40* overexpression enhances the susceptibility of native tobacco plants to *R. solanacearum*.

Consistent with the enhanced susceptibility of the *GhWRKY40-OE* plants, they also exhibited reduced transcript levels of the SA production-associated genes *PR1a* and *PR2* relative to the WT plants after *R. solanacearum* infection. Similarly, the transcript level of ET-responsive gene *ACS6* was decreased. After *R. solanacearum* infection, the JA-responsive genes *JAZ1* and *JAZ3* were lower in the OE plants than in the WT plants. The ROS detoxification-associated genes *APX*, *GST* and *SOD* exhibited increased transcript levels in the OE plants after *R. solanacearum* infection, and their transcripts accumulated to higher levels in the OE plants relative to the WT plants. Expression of the HR-associated gene *HIN1* was obviously induced, as its transcript accumulated to higher levels in the OE plants than in the WT plants. However, the transcript levels of *PR4* and *LOX1* did not show any significant difference in the OE plants relative to the WT plants (Fig. 6 B–C).

### GhWRKY40 interacts with GhMPK20 but not with GhMPK6a

**Figure 7:**
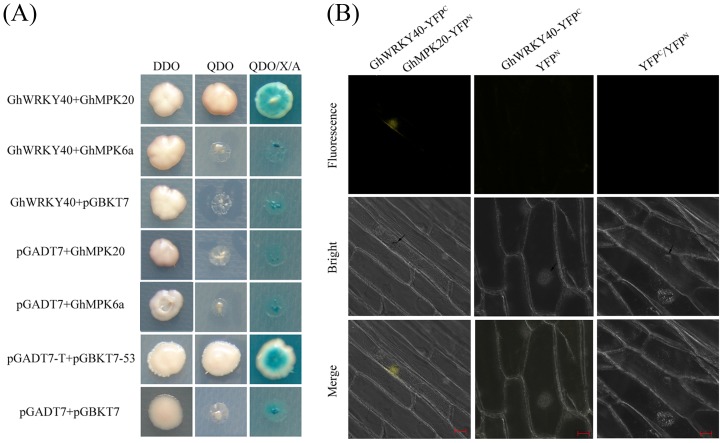
Interaction between GhWRKY40 and GhMPK20/GhMPK6a in yeast and onion epidermal cells. (A) Transformants grown on DDO, QDO or QDO/X/A. (B) *In vitro* BiFC analysis of the GhWRKY40 -interacting protein GhMPK20 in co-transformed into onion epidermal cells. The yellow fluorescence indicates the interaction between GhWRKY40 and GhMPK20. The fluorescent signals were observed using a confocal microscope. Scale bar = 20 µm.

Yeast two-hybrid and BiFC systems were used to determine the interactions between GhWRKY40 and GhMPK6a (HM055511)/GhMPK20 (HQ828072). In the yeast two-hybrid system, positive clones expressing GhMPK20 and GhWRKY40 were able to grow on QDO and QDO/X/A plates, indicating that GhWRKY40 interacted with GhMPK20 due to the activation of the reporter genes *AbA* and *MEL1*. However, yeast cells cotransformed with GhMPK6a and GhWRKY40 were unable to grow on QDO and QDO/X/A plates (Fig. 7A). These interactions were further confirmed with the BiFC system, in which two plasmids were constructed for the expression of GhWRKY40-yellow fluorescent protein (YFP)^C^ and GhMPK20-YFP^N^, reseparately, and then cotransformed into onion epidermal cells by particle bombardment. The YFP fluorescence signal in the onion epidermal cells transfected with GhWRKY40-YFP^C^ and GhMPK20-YFP^N^ was exclusively nuclear (Fig. 7B). Notably, GhMPK20 was detected in both the cytoplasm and nucleus (unpublished data). These data demonstrate that GhWKY40 interacts with GhMPK20 in the nucleus.

## Discussion

To gain an increased understanding of WRKY transcription factors in cotton, we cloned a *WRKY* gene from *G. hirsutum*. Our sequence and phylogenetic tree analyses indicated that the *GhWRKY40* gene belongs to subgroup IIa (Fig. 1B). Our subcellular localization experiment with GhWRKY40-GFP indicated that GhWRKY40 is located in the nucleus (Fig. 2B), which is consistent with previous studies on WRKY transcription factors from other species [29]. Moreover, consistent with the putative role of WRKY proteins as transcription factors, three nuclear targeting sequences were identified (Fig. 1A). Transcriptional activation analysis in yeast showed that the full-length GhWRKY40 protein is transcriptionally active (Fig. 2C). These results suggest that *GhWRKY40* is a member of the WRKY family in cotton and may serve as a transcriptional activator.

Because plants are sessile, they are constantly affected by their environmental conditions in the form of different abiotic and biotic stresses. Wounding is a common injury in plants that occurs as a result of abiotic stress factors, such as wind, rain and hail, and biotic factors, especially insect feeding. A previous microarray study that focused on the transcriptional profiling of genes after wounding indicated that wounding induces the expression of WRKY family proteins in *Arabidopsis* [16]. In this study, we found that the *GhWRKY40* transcript level is induced by wounding treatment (Fig. 3B) and that *GhWRKY40* overexpression affects the expression of defense-related genes in response to wounding (Fig. 5B). The transcriptional induction of *JAZ1* and *JAZ3* in wounded *GhWRKY40-OE* plants is lower than in wild-type plants, suggesting that *GhWRKY40* negatively controls the expression of certain *JAZ* genes. Jasmonate ZIM-domain (JAZ) genes, key repressors of JA signaling, are primary response genes in the JA signaling pathway. Most members of the JAZ gene family are highly expressed in response to mechanical wounding [30]. The promoters of *JAZ* genes contain several W boxes, which can be bound by WRKY genes. In addition, the overexpression of *GhWRKY40* was also found to significantly inhibit the expression of *PR* genes. *PR*s, defense-related genes often associated with SA-mediated defense responses. Moreover, the expression of *APX*, *SOD* and *GST* is slightly enhanced in response to wounding. *PR*s and *GST* are induced by wounding and have been identified as late response genes [16]. In addition, the ROS levels were lower in OE lines. Thus, the wounding tolerance of OE plants might be correlated with oxidative tolerance. Both wounding and pathogenic attack induce the expression of WRKYs, and the responses to wounding and pathogenic infection in plants share a number of signal transduction pathway components [31]. *PR*s and *JAZ*s have been reported to be such components. The expression of *PR*s and *JAZ*s were found to be significantly inhibited by the overexpression of *GhWRKY40* after *R. solanacearum* infection (Fig. 6C), and lead to the susceptibility of transgenic plants to *R. solanacearum*. Similarly, *AtWRKY40* affects JA signaling by directly controlling the expression of a subset of *JAZ*s upon plant-pathogen interaction, transcriptional reprogramming regulated by *WRKY40* facilitates powdery mildew infection of *Arabidopsis* [32]. Taken together, *GhWRKY40* may be a key component in response to wounding and *R. solanacearum* attack, and *JAZ*s and *PR*s may play roles in *GhWRKY40*-mediated crosstalk between wounding and pathogen defense responses.

As discussed earlier, the signaling molecules SA, JA and ET play important roles in the regulation of the complex defense mechanisms [33]. Previous studies have revealed that responses against biotrophic pathogens are generally regulated by SA, while responses to necrotrophs are mediated by JA and ET [34]–[35]. SA, JA and ET have been shown to activate different sets of plant *PR* genes and to act either synergistically or antagonistically during defense signaling [36]–[38]. In numerous plants, the transcription of WRKY genes is strongly and rapidly upregulated in response to pathogen infection or defense-related plant hormones, such as SA and JA. In cotton, *GhWRKY40* was upregulated by the *R. solanacearum*, MeJA and SA (Fig. 3). Moreover, the overexpression of *GhWRKY40* decreased the resistance of transgenic plants to *R. solanacearum* infection, as well as *PR* and *JAZ* gene transcripts (Fig. 6). SA accumulates in infected leaves after infection with biotrophic pathogens and mediates the induced expression of defense genes, resulting in an enhanced state of defense known as systemic acquired resistance (SAR) [39]. *PR*s are often used as molecular markers for SAR. In *Arabidopsis*, the enhanced susceptibility of transgenic plants overexpressing *WRKY8* to *Pseudomonas syringae* was associated with reduced expression of *PR1* [40]. Moreover, *Arabidopsis WRKY8* is a wounding-induced WRKY gene. In addition to their involvement in disease resistance signaling, SA, JA and ET have been reported to be involved in the wounding response [16], . We showed that *GhWRKY40* is transcriptionally inducible by wounding (Fig. 3) and that the overexpression of *GhWRKY40* represses the expression of the SA-dependent genes *PR1a*, *PR2* and *PR4* and the JA-responsive genes *JAZ1* and *JAZ3* upon wounding (Fig. 5). Therefore, we speculate that SA/JA induce *GhWRKY40* expression which leads to the reduction in expression of downstream defense genes.

WRKY TFs exhibit autoregulation and crossregulation activities and also interact with different proteins, such as MAP kinases, to carry out diverse plant functions [8], [42]. The last decade of research has shown that MPKs regulate WRKY TFs in response to multiple stresses. In previous studies, we functionally identified two MAPK genes from cotton, *GhMPK6a* [43] and *GhMPK20* (unpublished). The results obtained in the present study indicate that GhWRKY40 interacts with GhMPK20 but not with GhMPK6a (Fig. 7). In *Arabidopsis*, the group II WRKY proteins of WRKY6 and WRKY22 were found to interact with MPK10 and MPK3/MPK6, respectively [44], [42]. However, the identification of the upstream components that regulate WRKY TFs is difficult due to cellular interactions, redundancy, and functional pleiotropy. Our results provide valuable information that aids our understanding of the relationship between MAPKs and the WRKY family proteins in cotton and enhances our understanding of the molecular mechanism of signal transduction in cotton plants under stress.

In conclusion, our results suggest that *GhWRKY40* responds to a variety of stresses and that the overexpression of *GhWRKY40* in *N. benthamiana* affects defense-related gene express, enhances the resistance to wounding and the susceptibility to bacterial pathogen. Furthermore, we show that GhWRKY40 interacts with GhMPK20 both *in vivo* and *in vitro*. The elucidation of the regulatory mechanism of *GhWRKY40* overexpression may reveal a converging node in the regulatory pathways involved the plant responses to wounding and pathogenic infection. Understanding the biological function of *GhWRKY40* in cotton enriches our knowledge concerning WRKY function in crops. As we learn more about WRKY regulation, potential applications in genetic improvement should become possible in crops.

## Supporting Information

Figure S1 Comparison of the seed germination and post germination of WT and OE plants. (A) Seeds germination phenotype of WT and OE lines on MS medium. (B) The germination rate (greening cotyledon ratio) of the seeds under normal condition. Germination was scored daily. (C) The mass of thousand grains of WT and OE plants. (D) The fresh weight (weight of twenty seedlings) of the seedlings was recorded 10 d after sowing. The data shown indicate the means ± standard errors of three independent experiments. Different letters above the columns indicate significant differences (P<0.01) according to Duncan's multiple range test using SAS version 9.1 software.
(TIF)

Figure S2 The full-length cDNA sequence and primers on the sequence of *GhWRKY40*. The primers mentioned in the text were underlined. The initiation codon and termination codon was bold.
(TIF)

Table S1 Polymerase chain reaction amplification conditions.
(DOC)

Table S2 Primers used for gene cloning.
(DOC)

Table S3 The primers used for qPCR.
(DOC)

## References

[1] Mukhopadhyay A, Vij S, Tyagi AK (2004) Overexpression of zinc-finger protein gene from rice confers tolerance to cold, dehydration, and salt stress in transgentic tobacco. Proc Nat Acad Sci U S A 101: 6309–6314. 10.1073/pnas.0401572101 PMC395965 15079051

[2] Singh K, Foley RC, Onate-Sanchez L (2002) Transcription factors in plant defense and stress responses. Curr Opin Plant Biol 5: 430–436. 12183182 10.1016/s1369-5266(02)00289-3

[3] Vinocur B, Altman A (2005) Recent advances in engineering plant tolerance to abiotic stress: achievements and limitations. Curr Opin Plant Biol 16: 123–132. 10.1016/j.copbio.2005.02.001 15831376

[4] Yamaguchi-Shinozaki K, Shinozaki K (2006) Transcriptional regulatory networks in cellular responses and tolerance to dehydration and cold stresses. Annu Rev Plant Biol 57: 781–803. 16669782 10.1146/annurev.arplant.57.032905.105444

[5] Ren X, Chen Z, Liu Y, Zhang H, Zhang M, et al (2010) ABO3, a WRKY transcription factor, mediates plant responses to abscisic acid and drought tolerance in *Arabidopsis* . Plant J 3: 417–429. 10.1111/j.1365-313X.2010.04248.x PMC3117930 20487379

[6] Eulgem T, Rushton PJ, Robatzek S, Somssich IE (2000) The WRKY superfamily of plant transcription factors. Trends Plant Sci 5: 199–206. 10785665 10.1016/s1360-1385(00)01600-9

[7] Agarwal PK, Agarwal P, Reddy MK, Sopory SK (2006) Roles of DREB transcription factors in abiotic and biotic stress tolerance in plants. Plant Cell Rep 25: 1263–1274. 16858552 10.1007/s00299-006-0204-8

[8] Rushton PJ, Somssich IE, Ringler P, Shen QJ (2010) WRKY transcription factors. Trends in Plant Science 15: 247–258. 20304701 10.1016/j.tplants.2010.02.006

[9] Birkenbihl RP, Diezel C, Somssich IE (2012) Arabidopsis *WRKY33* is a key transcriptional regulator of hormonal and metabolic responses toward *Botrytis cinerea* infection. Plant Physiol 159 (1)266–285. 22392279 10.1104/pp.111.192641 PMC3375964

[10] Jiang Y, Deyholos MK (2009) Functional characterization of Arabidopsis NaCl-inducible WRKY25 and WRKY33 transcription factors in abiotic stresses. Plant Mol Biol 69 (1–2)91–105. 18839316 10.1007/s11103-008-9408-3

[11] Li S, Fu Q, Chen L, Huang W, Yu D (2011) Arabidopsis thaliana WRKY25, WRKY26, and WRKY33 coordinate induction of plant thermotolerance. Planta 233 (6)1237–1252. 21336597 10.1007/s00425-011-1375-2

[12] Dang FF, Wang YN, Yu L, Eulgem T, Lai Y, et al (2012) *CaWRKY40*, a WRKY protein of pepper, plays an important role in the regulation of tolerance to heat stress and resistance to *Ralstonia solanacearum* infection. Plant Cell Environ 36 (4)757–774. 22994555 10.1111/pce.12011

[13] Chen W, Provart N, Glazebrook J, Katagiri F, Chang HS, et al (2002) Expression profile matrix of Arabidopsis transcription factor genes suggests their putative functions in response to environmental stresses. Plant Cell 14: 559–574. 11910004 10.1105/tpc.010410 PMC150579

[14] Maleck K, Levine A, Eulgem T, Morgan A, Schmid J, et al (2000) The transcriptome of *Arabidopsis thaliana* during systemic acquired resistance. Nat Genet 26: 403–409. 11101835 10.1038/82521

[15] Schenk PM, Kazan K, Wilson I, Anderson JP, Richmond T, et al (2000) Coordinated plant defense responses in *Arabidopsis* revealed by microarray analysis. Proc Natl Acad Sci U S A 97: 11655–11660. 11027363 10.1073/pnas.97.21.11655 PMC17256

[16] Cheong YH, Chang HS, Gupta R, Wang X, Zhu T, et al (2002) Transcriptional profiling reveals novel interactions between wounding, pathogen, abiotic stress, and hormonal responses in *Arabidopsis* . Plant Physiol 129 (2)661–677. 12068110 10.1104/pp.002857 PMC161692

[17] Asai T, Tena G, Plotnikova J, Willmann MR, Chiu WL, et al (2002) MAP kinase signalling cascade in *Arobidopisis* innate immunity. Nature 415: 977–983. 11875555 10.1038/415977a

[18] Nakagami H, Soukupova H, Schikora A, Zarsky V, Hirt H (2006) A mitogen-activated protein kinase kinase kinase mediates reactive oxygen species homeostasis in *Arabidopsis* . J Biol Chem 281: 38697–38704. 17043356 10.1074/jbc.M605293200

[19] Ishihama N, Yamada R, Yoshioka M, Katou S, Yoshioka H (2011) Phosphorylation of the *Nicotiana benthamiana* WRKY8 transcription factor by MAPK functions in the defense response. Plant Cell 23: 1153–1170. 21386030 10.1105/tpc.110.081794 PMC3082260

[20] Shen H, Liu C, Zhang Y, Meng X, Zhou X, et al (2012) OsWRKY30 is activated by MAP kinases to confer drought tolerance in rice. Plant Mol Biol 80 (3)241–253. 22875749 10.1007/s11103-012-9941-y

[21] Wang X, Xiao H, Chen G, Zhao X, Huang C, et al (2011) Isolation of high-quality RNA from *Reaumuria soongorica*, a desert plant rich in secondary metabolites. Mol Biotechnol 48: 165–172. 21136208 10.1007/s12033-010-9357-3

[22] Yu F, Huaxia Y, Lu W, Wu C, Cao X, et al (2012) *GhWRKY15*, a member of the WRKY transcription factor family identified from cotton (*Gossypium hirsutum* L.), is involved in disease resistance and plant development. BMC Plant Biol 12: 144. 22883108 10.1186/1471-2229-12-144 PMC3489871

[23] Horsch RB, Rogers SG, Fraley RT (1985) Cold Spring Harbor Symposia on Quantitative Biology. Transgenic plants 50: 433–437. 10.1101/sqb.1985.050.01.054 3868487

[24] Fujita M, Fujita Y, Noutoshi Y, Takahashi F, Narusaka Y, et al (2006) Crosstalk between abiotic and biotic stress responses: a current view from the points of convergence in the stress signaling networks. Curr Opin Plant Biol 9 (4)436–442. 16759898 10.1016/j.pbi.2006.05.014

[25] León J, Rojo E, Sanchez-Serrano JJ (2001) Wounding signaling in plants. J Exp Bot 52 (354)1–9. 10.1093/jexbot/52.354.1 11181708

[26] O'Donnell PJ, Calvert C, Atzorn R, Wasternack C, Leyser HMO, et al (1996) Ethylene as a signal mediating the wound response of tomato plants. Science 274: 1914–1917. 8943205 10.1126/science.274.5294.1914

[27] Ecker JR (1995) The ethylene signal transduction pathway in plants. Science 268 (5211)667–675. 7732375 10.1126/science.7732375

[28] Reymond P, Farmer EE (1998) Jasmonate and salicylate as global signals for defense gene expression. Curr Opin Plant Biol 1: 404–411. 10066616 10.1016/s1369-5266(98)80264-1

[29] Zhang CQ, Xu Y, Lu Y, Yu HX, Gu MH, et al (2011) The WRKY transcription factor *OsWRKY78* regulates stem elongation and seed development in rice. Planta 234 (3)541–554. 21547461 10.1007/s00425-011-1423-y

[30] Chung HS, Koo AJ, Gao X, Jayanty S, Thines B, et al (2008) Regulation and function of Arabidopsis JASMONATE ZIM-Domain genes in response to wounding and herbivory. Plant Physiol 146 (3)952–964. 18223147 10.1104/pp.107.115691 PMC2259048

[31] Du L, Chen Z (2000) Identification of genes encoding receptor-like protein kinases as possible targets of pathogen- and salicylic acid-induced WRKY DNA binding proteins in *Arabidopsis* . Plant J 24: 837–847. 11135117 10.1046/j.1365-313x.2000.00923.x

[32] Pandey SP, Roccaro M, Schön M, Logemann E, Somssich IE (2010) Transcriptional reprogramming regulated by *WRKY18* and *WRKY40* facilitates powdery mildew infection of *Arabidopsis* . Plant J 64 (6)912–923. 21143673 10.1111/j.1365-313X.2010.04387.x

[33] Spoel SH, Johnson JS, Dong X (2007) Regulation of tradeoffs between plant defenses against pathogens with different lifestyles. Proc Natl Acad Sci U S A 104: 18842–18847. 17998535 10.1073/pnas.0708139104 PMC2141864

[34] Vlot AC, Dempsey DMA, Klessig DF (2009) Salicylic acid, a multifaceted hormone to combat disease. Annual Review of Phytopathology 47: 177–206. 10.1146/annurev.phyto.050908.135202 19400653

[35] Farmer EE, Alméras E, Krishnamurthy V (2003) Jasmonates and related oxylipins in plant responses to pathogenesis and herbivory. Curr Opin Plant Biol 6: 372–378. 12873533 10.1016/s1369-5266(03)00045-1

[36] Leon-Reyes A, Du Y, Koorneef A, Proietti S, Körbes AP, et al (2010) Ethylene signaling renders the jasmonate response of *Arabidopsis* insensitive to future suppression by salicylic acid. Mol Plant-Microbe Interact 23: 187–197. 20064062 10.1094/MPMI-23-2-0187

[37] Mur LA, Kenton P, Atzorn R, Miersch O, Wasternack C (2006) The outcomes of concentration-specific interactions between salicylate and jasmonate signaling include synergy, antagonism, and oxidative stress leading to cell death. Plant Physiol 140: 249–262. 16377744 10.1104/pp.105.072348 PMC1326048

[38] Koornneef A, Pieterse CMJ (2008) Cross talk in defense signaling. Plant Physiol 146: 839–844. 18316638 10.1104/pp.107.112029 PMC2259093

[39] Glazebrook J (2005) Contrasting mechanisms of defense against biotrophic and necrotrophic pathogens. Annu Rev Phytopathol 43: 205–227. 16078883 10.1146/annurev.phyto.43.040204.135923

[40] Chen L, Zhang L, Yu D (2010) Wounding-induced *WRKY8* is involved in basal defense in *Arabidopsis* . Mol Plant Microbe Interact 23 (5)558–565. 20367464 10.1094/MPMI-23-5-0558

[41] Reymond P, Farmer EE (1998) Jasmonate and salicylate as global signals for defense gene expression. Curr Opin. Plant Biol 1: 404–411. 10.1016/s1369-5266(98)80264-1 10066616

[42] Popescu SC, Popescu GV, Bachan S, Zhang Z, Gerstein M, et al (2009) MAPK target networks in *Arabidopsis thaliana* revealed using functional protein microarrays. Genes Dev 1:23 (1)80–92. 10.1101/gad.1740009 PMC2632172 19095804

[43] Li Y, Zhang L, Wang X, Zhang W, Hao L, et al (2013) Cotton GhMPK6a negatively regulates osmotic tolerance and bacterial infection in transgenic *Nicotiana benthamiana*, and plays a pivotal role in development. FEBS J 280 (20)5128–5144. 23957843 10.1111/febs.12488

[44] Robatzek S, Somssich IE (2002) Targets of *AtWRKY6* regulation during plant senescence and pathogen defense. Genes Dev 16: 1139–1149. 12000796 10.1101/gad.222702 PMC186251

