# Supplementary figures and images for: GhWRKY40, a Multiple Stress-Responsive Cotton WRKY Gene, Plays an Important Role in the Wounding Response and Enhances Susceptibility to Ralstonia solanacearum Infection in Transgenic Nicotiana benthamiana
Source: PLoS One. 2014 Apr 18;9(4):e93577. doi: 10.1371/journal.pone.0093577 (PMC3991585; doi:10.1371/journal.pone.0093577)

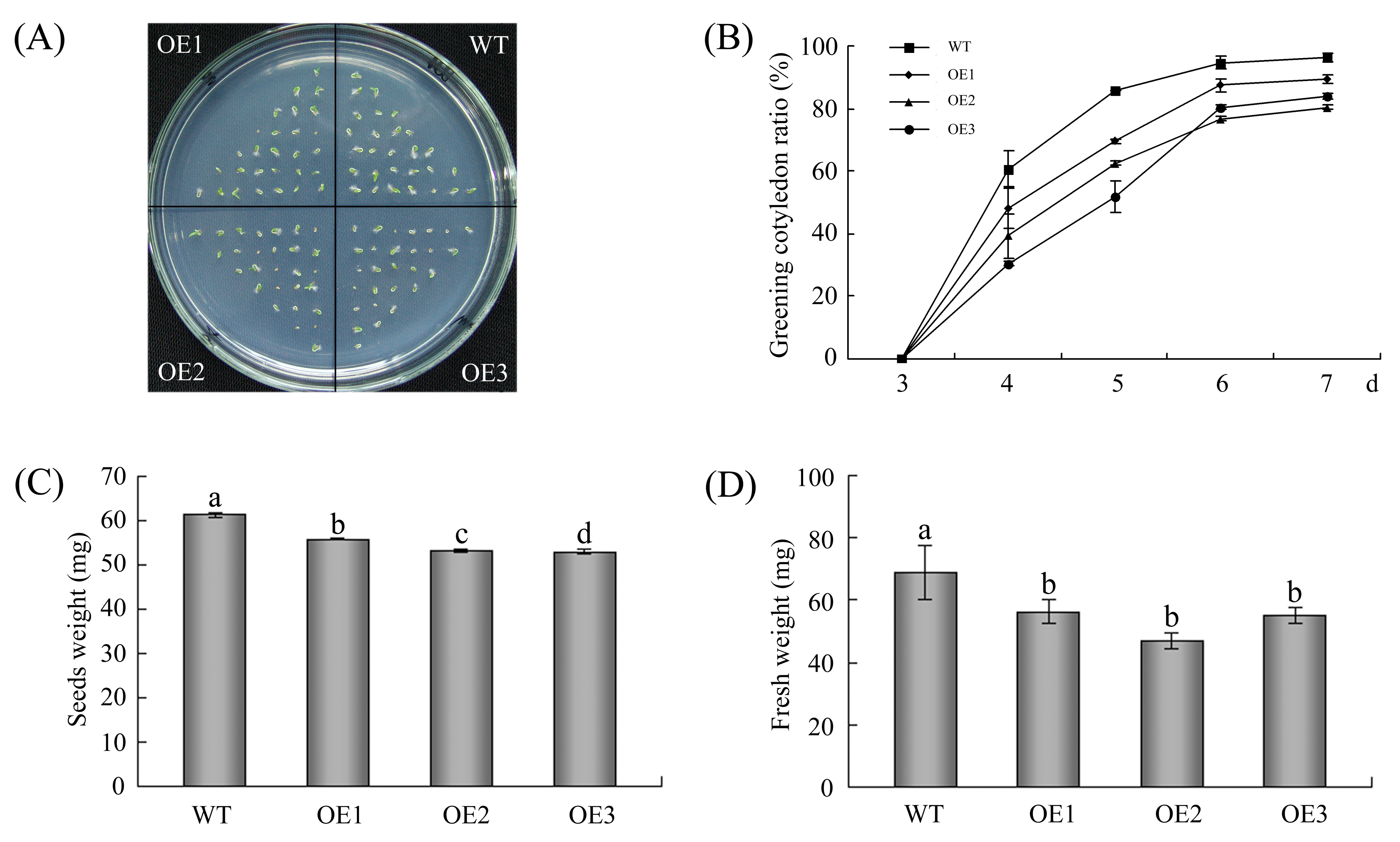

Supplement: Figure S1 — Comparison of the seed germination and post germination of WT and OE plants. (A) Seeds germination phenotype of WT and OE lines on MS medium. (B) The germination rate (greening cotyledon ratio) of the seeds under normal condition. Germination was scored daily. (C) The mass of thousand grains of WT and OE plants. (D) The fresh weight (weight of twenty seedlings) of the seedlings was recorded 10 d after sowing. The data shown indicate the means ± standard errors of three independent experiments. Different letters above the columns indicate significant differences (P<0.01) according to Duncan's multiple range test using SAS version 9.1 software. (TIF) [file pone.0093577.s001.tif]

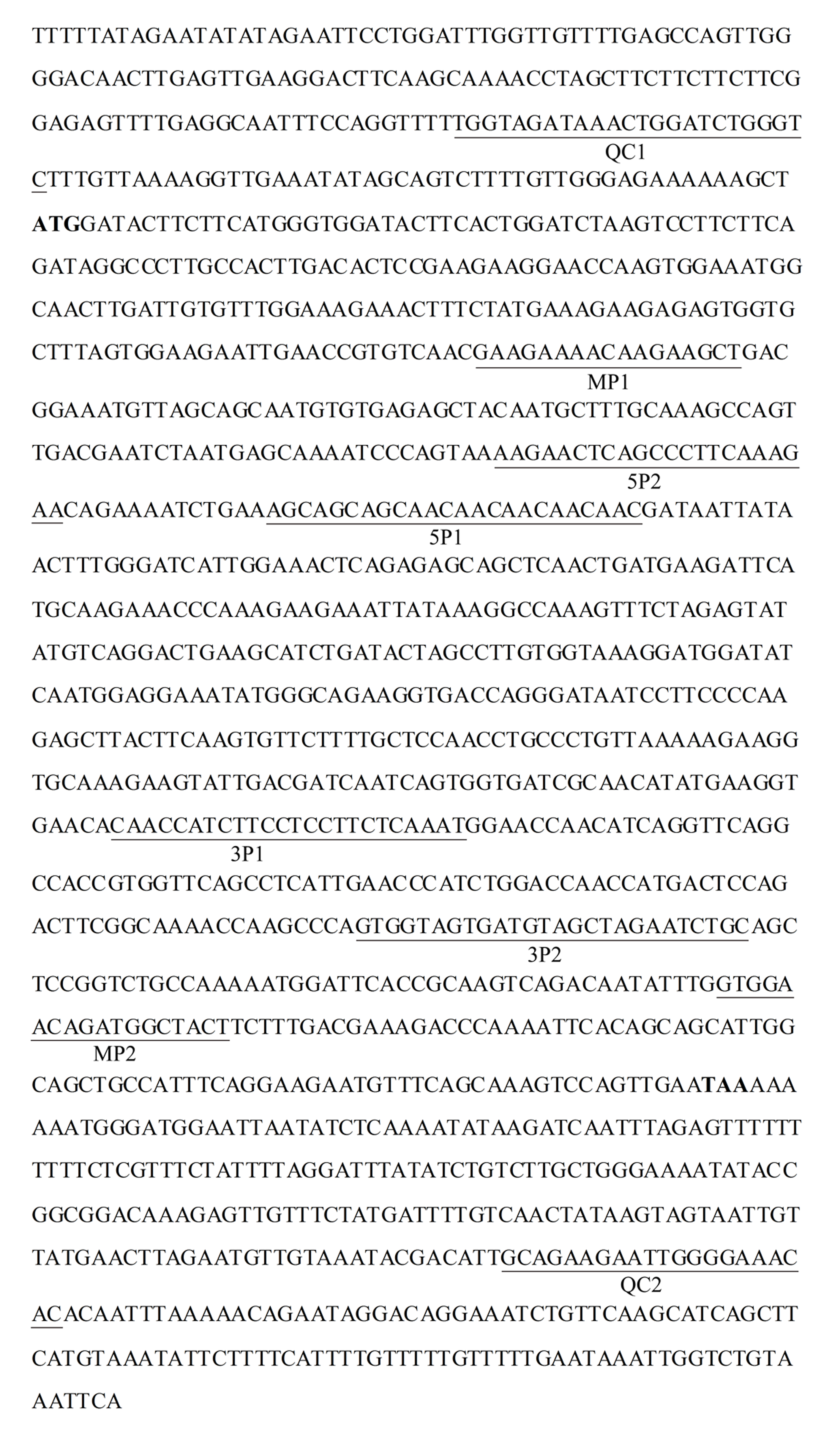

Supplement: Figure S2 — The full-length cDNA sequence and primers on the sequence of GhWRKY40. The primers mentioned in the text were underlined. The initiation codon and termination codon was bold. (TIF) [file pone.0093577.s002.tif]
