# Supplementary material for: GhWRKY40, a Multiple Stress-Responsive Cotton WRKY Gene, Plays an Important Role in the Wounding Response and Enhances Susceptibility to Ralstonia solanacearum Infection in Transgenic Nicotiana benthamiana
Source: PLoS One. 2014 Apr 18;9(4):e93577. doi: 10.1371/journal.pone.0093577 (PMC3991585; doi:10.1371/journal.pone.0093577)
Supplement: Table S1 — Polymerase chain reaction amplification conditions. (DOC) [file pone.0093577.s003.doc]

**Supplementary Table 1.** Polymerase chain reaction amplification conditions.

| Primers pair | PCR amplification conditions |
| --- | --- |
| MP1/MP2 | 94 °C for 10 min, 35 cycles of 94 °C for 40 s, 50 °C for 40 s and 72 °C for 1 min, then 72 °C for 10 min |
| 5P1/AAP | 94 °C for 5 min, 32 cycles of 94 °C for 40 s, 49 °C for 40 s and 72 °C for 1 min, then 72 °C for 10 min |
| 5P2/AUAP | 94 °C for 5 min, 35 cycles of 94 °C for 30 s, 50 °C for 30 s and 72 °C for 30 s, then 72 °C for 10 min |
| 3P1/B26 | 94 °C for 5 min, 32 cycles of 94 °C for 40 s, 51 °C for 40 s and 72 °C for 1 min, then 72 °C for 10 min |
| 3P2/B25 | 94 °C for 5 min, 35 cycles of 94 °C for 30 s, 52 °C for 30 s and 72 °C for 30 s, then 72 °C for 10 min |
| QC1/QC2 | 94 °C for 10 min, 35 cycles of 94 °C for 40 s, 50 °C for 40 s and 72 °C for 1 min, then 72 °C for 10 min |
| QG1/QG2 | 94 °C for 10 min, 35 cycles of 94 °C for 40 s, 50 °C for 40 s and 72 °C for 2 min , then 72 °C for 10 min |
| Nde1/Nde2  Nde1/Nde2 | 94 °C for 10 min, 32 cycles of 94 °C for 40 s, 49 °C for 40 s and 72 °C for 1 min 30 s, then 72 °C for 5 min  94 °C for 10 min, 35 cycles of 94 °C for 40 s, 51 °C for 40 s and 72 °C for 1 min , then 72 °C for 5 min |
| Ssp1/Ssp2  Ssp1/Ssp2 | 94 °C for 10 min, 32 cycles of 94 °C for 40 s, 49 °C for 40 s and 72 °C for 1 min 30 s, then 72 °C for 5 min  94 °C for 10 min, 35 cycles of 94 °C for 40 s, 52 °C for 40 s and 72 °C for 1 min , then 72 °C for 5 min |
| Vsp1/Vsp2  Vsp1/Vsp2 | 94 °C for 10 min, 32 cycles of 94 °C for 40 s, 49 °C for 40 s and 72 °C for 1 min 30 s, then 72 °C for 5 min  94 °C for 10 min, 35 cycles of 94 °C for 40 s, 51 °C for 40 s and 72 °C for 1 min , then 72 °C for 5 min |
| WP1/WP2 | 94 °C for 5 min, 35 cycles of 94 °C for 40 s, 50 °C for 40 s and 72 °C for1 min, then 72 °C for 10 min |
