# Supplementary material for: GhWRKY40, a Multiple Stress-Responsive Cotton WRKY Gene, Plays an Important Role in the Wounding Response and Enhances Susceptibility to Ralstonia solanacearum Infection in Transgenic Nicotiana benthamiana
Source: PLoS One. 2014 Apr 18;9(4):e93577. doi: 10.1371/journal.pone.0093577 (PMC3991585; doi:10.1371/journal.pone.0093577)
Supplement: Table S2 — Primers used for gene cloning. (DOC) [file pone.0093577.s004.doc]

**Supplementary Table 2.** Primers used for gene cloning.

| Abbreviation | Primer sequence (5′-3′) | Description |
| --- | --- | --- |
| MP1 | GCHGARAACAAGAAGCT | cDNA sequence primer, forward |
| MP2 | AGTRGCCATYTGYTCCAC | cDNA sequence primer, reverse |
| 5P1 | AGCAGCAACAACAACAACAAC | 5′ RACE reverse primer, outer |
| 5P2 | AAGAACTCAGCCCTTCAAAGAA | 5′ RACE reverse primer, inner |
| AAP | GGCCACGCGTCGACTAGTAC(G)_14_ | Abridged anchor primer |
| AUAP | GGCCACGCGTCGACTAGTAC | Abridged universal amplification primer |
| 3P1 | CAACCATCTTCCTCCTTCTCAAAT | 3′ RACE forward primer, outer |
| 3P2 | GTGGTAGTGATGTAGCTAGAATCTGC | 3′ RACE forward primer, inner |
| B26 | GACTCTAGACGACATCGA(T)_18_ | 3′ RACE universal adaptor primer |
| B25 | GACTCTAGACGACATCGA | 3′ RACE universal primer |
| QC1 | TGGTAGATAAACTGGATCTGGGTC | Full-length cDNA primer, forward |
| QC2 | GCAGAAGAATTGGGGAAACAC | Full-length cDNA primer, reverse |
| QG1 | TGGTAGATAAACTGGATCTGGGTC | Genomic sequence primer, forward |
| QG2 | GCAGAAGAATTGGGGAAACAC | Genomic sequence primer, reverse |
| Nde1 | GATGTACCGGAGTGTCAAGT | Inverse PCR forward primer, outer |
| Nde2 | GAAGGAACCAAGTGGAAAT | Inverse PCR reverse primer, outer |
| Nde3 | TGAAGTATCCACCCATGAAGAAGT | Inverse PCR forward primer, inner |
| Nde4 | TGAAAGAAGAGGTGAGTTTATGC | Inverse PCR forward primer, inner |
| Ssp1 | GATGTACCGGAGTGTCAAGT | Inverse PCR forward primer, outer |
| Ssp2 | CACAACCATCTTCCTCCTTCT | Inverse PCR reverse primer, outer |
| Ssp3 | TGAAGTATCCACCCATGAAGAAGT | Inverse PCR forward primer, inner |
| Ssp4 | GACCAACCATGACTCCAGACT | Inverse PCR reverse primer, inner |
| Vsp1 | GATGTACCGGAGTGTCAAGT | Inverse PCR forward primer, outer |
| Vsp2 | CACAACCATCTTCCTCCTTCT | Inverse PCR reverse primer, outer |
| Vsp3 | TGAAGTATCCACCCATGAAGAAGT | Inverse PCR forward primer, inner |
| Vsp4 | TCAGCCTCATTGAACCCATCT | Inverse PCR reverse primer, inner |
| WP1 | TACAATACGAATGGAATGAAGGT | Primer of the promoter |
| WP2 | AAAACCTGGAAATTGCCTCAAAC | Primer of the promoter |
