# Supplementary material for: GhWRKY40, a Multiple Stress-Responsive Cotton WRKY Gene, Plays an Important Role in the Wounding Response and Enhances Susceptibility to Ralstonia solanacearum Infection in Transgenic Nicotiana benthamiana
Source: PLoS One. 2014 Apr 18;9(4):e93577. doi: 10.1371/journal.pone.0093577 (PMC3991585; doi:10.1371/journal.pone.0093577)
Supplement: Table S3 — The primers used for qPCR. (DOC) [file pone.0093577.s005.doc]

**Supplementary Table 3.** The primers used for qPCR.

| Name | Accession no. | Primer sequence (5'-3') |
| --- | --- | --- |
| *GhWRKY40* | KC414679 | AACACAACCATCTTCCTCCTTCTC forward (WRT1) |
|  |  | TTTGCCGAAGTCTGGAGTCA reverse (WRT2) |
| *Ghubiquitin* | EU304080 | CCAGAAGGAATCCACTTTGC forward (Ub1) |
|  |  | CCAGCTCACATCAGCATACG reverse (Ub2) |
| *Nbβ-actin* | JQ256516.1 | TGGACTCTGGTGATGGTGTC forward |
|  |  | CCTCCAATCCAAACACTGTA reverse |
| *JAZ1* | ACY30445.1 | GTCACCGGCCAGAAGTCTC forward |
|  |  | TGGCACCTGAGTTCGCGTAC reverse |
| *JAZ3* | BAG68657.1 | CTGAGGCAAAATCTGAACCGGAG forward |
|  |  | GCACCCAATCCAAGCCACAC reverse |
| *LOX1* | X84040.1 | GTTGAAGGTTCTATCTGGCAGTTGG forward |
|  |  | TGTTGCGATCACGAATGGCTCTA reverse |
| *ACS6* | AF392978 | GCATTGTTATGAGTGGAGGGG forward |
|  |  | CAGATTCTAAGGCTTCTTTTGTGAC reverse |
| *APX* | U15933.1 | CGCTCCTCTTATGCTCCGTCTT forward |
|  |  | GGTGGCTCTGTCTTGTCCTCTC reverse |
| *SOD* | AB093097 | GGAGAGCCTTGTCTGATGG forward |
|  |  | TGGGTCCTGATTAGCAGTGGT reverse |
| *GST* | D10524 | AGCACCCTTACCTTTCCCTC forward |
|  |  | GCTTTCCTTCACAGCAGCATCA reverse |
| *PR1a* | X12485.1 | GGTGTAGAACCTTTGACCTGG forward |
|  |  | GAACCCTAGCACATCCAACAC reverse |
| *PR2* | M60460.1 | ACCATCAGACCAAGATGT forward |
|  |  | TGGCTAAGAGTGGAAGGT reverse |
| *PR4* | EH365959.1 | CAGAACATTAACTGGGATTTGAGAG forward |
|  |  | CTCCATTTGCTGCATTGATCTACT reverse |
| *HIN1* | Y07563 | CGACCTAACAAAGTCAAGTTCTACG forward |
|  |  | CTCTATCTCCCAATAAAACCAAGC reverse |
